# Is Targeting LDL-C Levels Below 70 mg/dL Beneficial for Cardiovascular and Overall Health? A Critical Examination of the Evidence

**DOI:** 10.3390/jcm14103569

**Published:** 2025-05-20

**Authors:** Folkert H. van Bruggen, David M. Diamond

**Affiliations:** 1 Department of Primary and Long-Term Care, University Medical Centre Groningen, University of Groningen, P.O. Box 196, 9700 AD Groningen, The Netherlands; 2 Department of Psychology, University of South Florida, Tampa, FL 33620, USA

**Keywords:** LDL-C targets, atherosclerosis, cardiovascular disease prevention, pleiotropic effects, ecological fallacy, lipid lowering therapy

## Abstract

Over the past two decades, cardiovascular disease (CVD) prevention guidelines have progressively lowered LDL-C targets to <70 mg/dL for high-risk individuals based on the assumption of a linear relationship between LDL-C levels and CVD risk. However, the available evidence challenges this premise. Multiple studies demonstrate a weak or inconsistent association between LDL-C levels and atherosclerosis progression at the individual patient-level. Systematic reviews supporting the linearity assumption have notable limitations, including extrapolation beyond observed LDL-C ranges and potential ecological fallacy, as meta-regression analyses rely on study-level data, while patient-level data within the same trials often show no association between LDL-C reduction and CVD outcomes. Moreover, randomized controlled trials explicitly designed to assess LDL-C targets have yielded inconclusive and biased results. LDL-C itself is a heterogeneous marker, with particle size and composition influencing its atherogenicity. The cardiovascular benefits of lipid-lowering therapies may arise in part from pleiotropic effects unrelated to LDL-C lowering. Additionally, several studies indicate that higher LDL-C levels are paradoxically associated with longevity in elderly populations that is equal to or even greater than that of the general population. Collectively, this body of evidence raises questions about the validity of current LDL-C targets < 70 mg/dL in high-risk patients.

## 1. Introduction

Over the past two decades, the strategy for managing cardiovascular disease (CVD) risk with lipid-lowering therapy has changed significantly. LDL-C targets in guidelines have been progressively lowered from <100 mg/dL (2.6 mmol/L) to 70 mg/dL (1.8 mmol/L) for high-risk patients and 55 mg/dL (1.4 mmol/L) for very high-risk patients [1]. The reduction in target LDL-C levels was stated as justified based on the appearance that intensive lipid-lowering therapy offered additional cardiovascular benefits compared to the standard regimens [2].

The establishment of low LDL-C targets in CVD prevention was based on the premise that there is a linear relationship between LDL-C levels and CVD risk [2]. However, this premise faces several challenges:

(1) The supposed direct correlation between LDL-C levels and atherosclerosis progression is questionable;
(2) The systematic reviews that provided the foundation for this assumption have several limitations, including extrapolation of results for LDL-C levels beyond observed data;
(3) Potential bias due to the ecological fallacy stemming from meta-regression results based on study-level rather than patient-level analyses;
(4) Inconsistent findings from trials specifically designed to investigate the relationship between LDL-C targets and CVD risk;
(5) Research documenting greater longevity of elderly individuals with familial—as well as non-familial—hypercholesterolemia contradicts the premise that lower LDL-C levels are ideal.

In this paper, we address these challenges point by point, providing evidence to support each argument. We also point out that LDL-C is a hybrid measure composed of heterogeneous particles, with varying atherogenicity depending on the size of the particles. Finally, we address evidence that pleiotropic effects of lipid-lowering therapies, particularly statins, may contribute to cardiovascular benefits, independent of LDL-C reduction. This paper, therefore, presents evidence to challenge current LDL-C targets < 70 mg/dL in patients at high CVD risk.

## 2. Challenging the Assumed Link Between LDL-C Reduction and Atherosclerotic Plaque Regression

The European Atherosclerosis Society (EAS) published a 2017 consensus statement demonstrating a linear relationship between LDL-C reduction and decreased atherosclerotic plaque volume, with the greatest reduction observed in patients with LDL-C levels < 70 mg/dL [3]. However, these findings were based on group-level analyses and introduced bias through the ecological fallacy, which occurs when group-level results are inappropriately applied to individual-level associations [4,5]. Inspection of the underlying studies, primarily post hoc analyses of randomized controlled trials (RCTs), revealed that six out of seven showed little to no correlation between LDL-C levels and plaque progression at the individual patient-level [6,7,8,9,10,11,12]. Additionally, a post hoc analysis of an RCT, along with another RCT published contemporaneously with the EAS statement, also failed to demonstrate this link [13,14]. Finally, Okuyama et al. addressed these issues, as well as other flaws in the EAS consensus statement [15].

The lack of association between LDL-C levels and plaque progression is revealed in another recent cohort study of 23,143 patients with symptoms suggestive of coronary artery disease that underwent coronary computed tomographic angiography [16]. The study reported an absence of a systematic relationship between any type of plaque (calcified or non-calcified) across different LDL-C strata.

These investigators also reported that coronary artery calcium (CAC) scores of 0, which excels at long-term risk prediction over periods of more than a decade [17,18,19], were not associated with LDL-C levels. Importantly, across all LDL-C strata, rates of atherosclerotic CVD and death were statistically equivalent and low in those with CAC scores of 0. Similarly, in patients treated with statins, the occurrence of coronary events was associated with higher CAC scores, but not with LDL-C levels [20].

These findings led Mortensen et al. to conclude “among symptomatic patients with high LDL-C levels (≥190 mg/dL) who are considered at universally high risk for ASCVD in guidelines with low LDL-C goals, absence of calcified and noncalcified coronary plaque was associated with very low event rates. These results highlight the multifactorial character of atherosclerosis as a disease not only driven by LDL-C levels …” [16]. Overall, these findings indicate that LDL-C levels serve as a weak indicator of atherosclerotic disease risk.

## 3. Lack of Association Between LDL-C Levels and Cardiovascular Events

The EAS guideline stated that “the greater the absolute LDL-C reduction, the greater the cardiovascular risk reduction”, based on the results of four systematic reviews [21,22,23,24]. However, these reviews have several important limitations.

First, the systematic reviews supporting this claim included only a limited number of trials achieving mean LDL-C levels < 70 mg/dL. Consequently, this target is largely based on extrapolation of study results, rather than direct evidence. Second, all four systematic reviews conducted meta-regression using aggregated trial-level data, despite two having access to individual patient-level data [21,24]. The latter would have allowed for an individual participant meta-analysis, enabling a direct assessment of the association between LDL-C and cardiovascular outcomes at patient-level that may differ from study-level [25]. Their approach may have introduced bias due to the ecological fallacy, a common issue in meta-regression that can lead to invalid conclusions [26]. Importantly, numerous primary prevention trials, including WOSCOPS, AFCAPS, MEGA, LRC-CPPT, CARDS, ALLHAT, and JUPITER, assessed LDL-C levels in relation to cardiovascular events. None of these trials found a linear relation between LDL-C levels and CVD risk [27,28,29,30,31,32,33]. Similar findings were observed in secondary or mixed prevention trials including CARE, LIPID, PROSPER, and SPARCL, where the relationship between LDL-C and cardiovascular outcomes was weak or non-linear [34,35,36,37]. Furthermore, a patient-level meta-analysis of PROMINENT, REDUCE-IT, and STRENGTH trials evaluating statin therapy in high-risk patients found no difference in major adverse cardiovascular events (MACE) between the lowest LDL-C quartile (range < 56 to <60 mg/dL) and higher LDL-C quartiles (range > 89 to >102 mg/dL) [38]. Similarly, an analysis of the REAL-CAD trial with a mixed prevention population found no significant correlation between on-treatment LDL-C levels below 70 mg/dL and reduced cardiovascular event risk compared to LDL-C levels of 70–100 mg/dL [39].

Third, most trials included in these systematic reviews were not designed to assess the relation between LDL-C targets and CVD risk. Aligning efficacy results with a pre-established relationship between clinical efficacy and LDL-C reduction may introduce observational bias [40,41]. Therefore, meta-regression analyses of such RCTs to demonstrate an association between LDL-C lowering and clinical outcomes should be approached cautiously, and their results considered observational [42]. Finally, a recent systematic review and an umbrella review of RCTs on statin efficacy found that LDL-C either showed no clear association with treatment effect size or failed as a trial-level surrogate for predicting all-cause mortality and cardiovascular events [43,44].

Overall, the lack of association between LDL-C and coronary events, which has been covered in detail elsewhere [45,46,47], provides strong evidence against the recommendation that “lower LDL is better” [48].

## 4. Inconsistent Clinical Outcomes and Bias in Trials with a Specific LDL-C Target

Three trials have been specifically designed to investigate the relationship between LDL-C targets and CVD risk. The results of these trials were inconsistent, failing to provide conclusive evidence for a clear association between specific LDL-C targets and the risk of CVD. The EMPATHY trial compared intensive statin therapy (LDL-C target < 70 mg/dL) to standard therapy (LDL-C target 100–120 mg/dL) in 5042 patients with hypercholesterolemia and diabetic retinopathy but no history of coronary artery disease. After a mean follow-up of 37 ± 13 months, the intensive group achieved a mean LDL-C of 76.5 mg/dL compared to 104.1 mg/dL in the standard group, but there was no statistically significant difference in the primary composite endpoint of cardiovascular events between groups [49].

Similarly, the HIJ-PROPER trial compared LDL-C targets of <70 mg/dL and <100 mg/dL in 1734 patients with acute coronary syndrome. During follow-up, mean LDL-C levels were 65.1 mg/dL in the intensive group versus 84.6 mg/dL in the control group. After a median follow-up of 3.9 years, there was no statistically significant difference in the primary composite endpoint (MACE) between groups [50].

The TST trial investigated LDL-C target levels and stroke risk in 2860 patients with prior stroke or transient ischemic attack (TIA). Patients were randomized to LDL-C targets of ≤70 mg/dL or 90–110 mg/dL. The trial was terminated early after 277 of the expected 385 end-point events had occurred. The primary composite endpoint (MACE) was observed in 8.5% of the lower-target group and 10.9% of the higher-target group (adjusted HR 0.78; 95% CI 0.61–0.98) [51]. However, several limitations in this trial raise concerns about the validity of its results. This study enrolled only 2873 patients, instead of the intended 3760 participants outlined in the protocol. This incomplete enrolment may have compromised the study’s statistical power and generalizability. Furthermore, a high dropout rate of 29.7% was observed, potentially introducing selection bias and affecting the accuracy of the estimated treatment effect. Also, the reliability of the primary outcome was questionable due to its low fragility index of 3, indicating that a shift in outcome for just three patients could have negated the statistical significance of the results [52]. This is especially concerning given that 141 patients were lost to follow-up. Lastly, the early termination of the trial may have led to an overestimation of treatment effects, a common issue in truncated studies [53].

## 5. Heterogeneity of LDL-C Particles

LDL-C is a hybrid measure composed of heterogeneous LDL particles which vary in size, density, and chemical composition [54]. The distinction between LDL particle subclasses based on size and density is important because the small dense LDL (sdLDL) particle is a component of the atherogenic dyslipidemia risk triad, composed of elevated levels of triglycerides (TGs) and sdLDL, in concert with low HDL-C [55,56,57]. High TGs, elevated sdLDL, and low HDL-C are each, individually, strong markers of CVD risk [54,58,59,60,61,62,63,64,65,66,67,68]. Conversely, large buoyant LDL (lbLDL) has not been shown to be a CVD risk factor [69,70,71]. Furthermore, only a moderate association between LDL-C levels and sdLDL-C levels has been reported [72]. Therefore, assessment of sdLDL and lbLDL subpopulations provides a greater prediction of CVD risk than does LDL-C [68].

In addition, oxidized LDL (ox-LDL) [54] and, more generally, measures of oxidative stress [73] are significant risk factors for atherosclerosis. Importantly, there is typically no correlation between LDL-C levels measured by traditional methods and the level of oxidized biomarkers [73] or oxidized LDL-C [74]. Similarly, for apolipoprotein B (apoB), which is considered a superior marker of lipid-related risk compared to LDL-C, there is a substantial variation in apoB values for any given LDL-C level [75].

Lipoprotein(a) [Lp(a)], a variant of LDL which is included in an LDL-C assay, is an independent risk factor for CVD [2]. Elevated Lp(a) can significantly affect LDL-C measurements, potentially leading to an overestimation of LDL-C levels [76]. A meta-analysis of five statin trials involving 18,043 patients demonstrated that CVD risk varied across LDL-C quartiles only when Lp(a) levels were factored into the measurement. After adjusting for Lp(a), the differences in risk across LDL-C quartiles disappeared [77]. Furthermore, in participants in lipid lowering trials that achieved LDL-C levels below 70 mg/dL, elevated Lp(a), but not LDL-C, predicted the CVD risk [78,79,80].

## 6. Pleiotropic Effects of Lipid-Lowering Therapy

The pleiotropic effects of lipid-lowering therapy may contribute to the inconsistent and non-linear association between LDL-C and risk of CVD. Independent of LDL-C lowering, statins reduce inflammation and have anticoagulant effects [81,82]. Baseline low-grade inflammation, measured by high-sensitivity C-reactive protein (hsCRP), has been shown to predict first cardiovascular events, even among patients on statin therapy [38,83]. A recent meta-analysis of 31,245 patients across three statin trials demonstrated distinct associations between inflammatory risk and residual cholesterol risk with cardiovascular outcomes among statin users. Higher baseline hsCRP levels were significantly associated with increased incidence of MACE. Notably, the relationship between LDL-C levels and MACE was neutral across LDL quartiles (LDL-C range < 56 to >102 mg/dL) [38]. In addition, statins reduce C-reactive protein (CRP) and hs-CRP levels in patients with CVD, independent of LDL-C levels [84]. Therefore, lower post-treatment CRP levels are associated with reduced CVD event risk, regardless of achieved LDL-C levels in primary and secondary prevention [85,86].

Statins also exhibit antithrombotic effects, directly and indirectly influencing coagulation and platelet aggregation [87]. In a secondary endpoint analysis of the JUPITER trial that compared rosuvastatin 20 mg daily versus placebo, rosuvastatin reduced the risk of venous thromboembolism (VTE). Importantly, the reduction in VTE risk was independent of the baseline LDL-C level [88]. Similar to previous trials comparing anticoagulant and antiplatelet drug combinations, rosuvastatin’s antithrombotic properties may explain its observed reduction in CVD risk [89,90].

Evolocumab and alirocumab lowered Lp(a) in addition to LDL-C. In the FOURIER and ODYSSEY OUTCOMES trials, these PCSK9 inhibitors achieved remarkably low LDL-C levels (30 and 38 mg/dL, respectively), while also significantly reducing Lp(a) levels [91,92]. Notably, the greatest reductions in MACE were observed in patients with higher baseline Lp(a) levels, rather than LDL-C levels. A post hoc analysis of the ODYSSEY OUTCOMES trial provided additional insight into the interplay between LDL-C and Lp(a). In patients with a baseline LDL-C level < 70 mg/dL, the treatment benefit of alirocumab was only observed in those with Lp(a) levels above 13.7 mg/dL. This benefit disappeared in patients with Lp(a) levels below 13.7 mg/dL, despite similarly low LDL-C levels [93].

Ezetimibe added to simvastatin significantly reduces both the atherogenic TGs and hs-CRP levels compared to simvastatin monotherapy [94]. This was demonstrated in the IMPROVE-IT trial that compared ezetimibe + simvastatin against simvastatin + placebo in patients at high risk of CVD. After one year of follow-up, there was a reduction in TGs (mean of 120.4 mg/dL compared to 137.1 mg/dL) and hs-CRP levels (3.3 mg/dL versus 3.8 mg/dL) in the intervention versus control group. Furthermore, among patients with LDL-C < 70 mg/dL, a significantly larger proportion also achieved hs-CRP < 2.0 mg/dL after one month in the ezetimibe + statin group (50.6%) compared to the simvastatin monotherapy group (30.5%). The authors of the IMPROVE-IT trial observed that the reduction in cardiovascular risk may not be solely attributable to LDL-C lowering, stating, “this trial cannot prove that the effect was mediated by the lowering of LDL cholesterol levels alone, since changes in other lipoproteins and high-sensitivity C-reactive protein may have played a role”. However, they did not further analyze whether the cardiovascular benefit would persist after adjusting for reductions in TGs and hs-CRP, which is necessary to substantiate their claim that lowering LDL-C levels alone provides cardiovascular benefit. Moreover, despite its LDL-C-lowering effects, ezetimibe did not reduce the risk of cardiovascular disease in patients without diabetes. An analysis paper of the IMPROVE-IT trial reported that among 13,209 patients without diabetes, after 7 years of follow-up, the hazard ratio (HR) for major adverse cardiovascular events (MACE) was 0.98 (95% CI 0.91–1.04), despite a mean LDL-C of 51 mg/dL (1.3 mmol/L) in the ezetimibe group versus 68 mg/dL (1.8 mmol/L) in the placebo group [95].

## 7. Hypercholesterolemia, Longevity, and Immune Function

An essential feature of the concept that “lower LDL is better” is based on the long-held view that hypercholesterolemia, whether based on genetics (familial hypercholesterolemia; FH) [3] or induced by diet [96,97,98,99,100,101], promotes CVD and premature all-cause mortality. However, numerous studies have demonstrated that elderly people with FH, as well as non-FH individuals, with extremely high LDL-C can live long, CVD-free lives [102,103,104,105,106], including no greater rate of cerebrovascular disease and ischemic stroke compared to the general population [107]. Indeed, Mundal et al. [102] reported that FH individuals in their 70s had significantly lower all-cause mortality compared to the age-matched general population, attributed largely to a normal rate of CVD death and extremely low rates of non-CVD death.

In contrast to elderly FH people, younger FH individuals are at an increased risk of developing CVD [102]. Their increased risk of CVD does not appear to be their high LDL-C since only a subset develops CVD. The basis of their CVD risk appears to be a hypersensitivity of their platelets to aggregating agents, e.g., adrenaline [108], and increased clotting factors, e.g., fibrinogen [109], which promotes thrombosis in a subset of FH individuals [110,111,112].

The salutary effects of higher LDL-C levels appear to be related to superior immune functioning [113,114], resistance to severe infection, such as sepsis, and protection from cancer-related mortality afforded to individuals with the highest LDL-C [115,116,117]. The high quality of health and longevity in elderly people with high LDL-C is incompatible with the consensus recommendation that lower LDL-C is healthier.

## 8. Discussion and Conclusions

Clinical guidelines have progressively lowered the recommended LDL-C target for high-risk CVD patients to below 70 mg/dL, but scientific support for this target is arguably questionable as summarized in Table 1. A rigorous assessment of the clinical findings based on both the results of numerous analysis papers of RCTs and some observational studies does not support the purported linear relationship between LDL-C levels and CVD risk, challenging the promotion of aggressive LDL-C reduction. Achieving these targets often requires intensive lipid-lowering therapy [118], which has shown limited clinical benefit [44,119] and raises significant safety concerns, including increased risk of new-onset type 2 diabetes [86,120,121,122,123,124,125] and increased fasting blood glucose [126], tendinopathy [127], myopathy [128,129], and cognitive deficits [130,131,132,133,134,135,136,137,138]. With regard to the latter, two ongoing RCTS—STAREE (NCT02099123) and PREVENTABLE (NCT04262206)—are currently investigating whether atorvastatin 40 mg, compared to placebo, can reduce the incidence of dementia and other outcomes in older adults without established cardiovascular disease. These trials may offer important insights into the potential impact of statin therapy on cognitive decline [139].

Studies have also shown that low LDL-C levels are associated with an increased risk of infections, sepsis, and mortality [117,140,141,142,143], while higher lipid levels may have a protective effect against infections [144,145] and infection-related mortality [140]. Also, observational cohort studies reported a U-shaped association between LDL-C- or total cholesterol levels and the risk of all-cause mortality, with the lowest mortality risk observed at LDL-C levels ranging between 100 and 189 mg/dL (2.6–4.0 mmol/L) [47,146,147,148,149]. This increased risk of mortality appears to be particularly evident in in patients with diabetes, regardless of whether they are at low or high cardiovascular risk. Results from the recently published ELSA-Brasil cohort revealed a U-shaped association between LDL-C levels and all-cause mortality in diabetic adults not at high cardiovascular risk, with increased mortality risk at LDL-C < 70 mg/dL (1.8 mmol/L) compared to levels between 100 and 129 mg/dL (2.6–3.3 mmol/L). The risk was even higher for those with LDL-C < 55 mg/dL (1.4 mmol/L) [150]. Complementary findings from the FOURIER trial suggest that very low LDL-C levels (average LDL-C level 31 mg/dL [0.8 mmol/L]) may similarly elevate all-cause mortality risk in diabetic patients with high cardiovascular risk [151,152,153].

Taken together, our review of the literature indicates that there is insufficient justification, and a strong need for caution, in pursuing extremely low LDL-C levels for the reduction of CVD risk. Instead, future research should focus on other lipid-related markers such as Lp(a). The ongoing OCEAN (olpasiran) and HORIZON (pelacarsen) trials will be crucial in determining whether Lp(a)-lowering therapies provide a cardiovascular benefit.

## Figures and Tables

**Table 1:** Summary of evidence challenging LDL-C target < 70 mg/dL.

Argument	Details
Lack of linear association between LDL-C and risk of CVD	- Minimal to no correlation has been observed between LDL-C levels and plaque progression or CAC scores at the individual patient level. - No consistent association has been found between LDL-C levels and cardiovascular events at the individual level. - Clinical trials targeting specific LDL-C levels have yielded inconsistent results regarding cardiovascular outcomes.
LDL-Cis a poor predictor of CVD risk	- LDL-C consists of diverse particles, with small dense LDL (sdLDL) strongly linked to CVD risk, while large buoyant LDL (lbLDL) is not. - Oxidized-LDL and Lp(a) are more reliable predictors of atherosclerosis and CVD. - Elevated Lp(a) can distort LDL-C measurements, and studies indicate that CVD risk in individuals with LDL-C < 70 mg/dL is primarily driven by high Lp(a) rather than LDL-C itself.
Lipid-lowering therapy benefits stem from reduction in non-LDL-C factors	The pleiotropic effects of lipid-lowering therapies, including the anti-inflammatory and antithrombotic actions of statins, the anti-inflammatory effects of ezetimibe, and the Lp(a)-lowering effect of PCSK9 inhibitors, contribute to cardiovascular risk reduction independently of LDL-C, with inflammation and Lp(a) being stronger determinants of risk.
Lifelong elevated LDL-C may result in long CVD-free lives	Elderly individuals with high LDL-C, including those with familial hypercholesterolemia (FH), often enjoy longer, CVD-free lives, likely due to enhanced immune function and resistance to severe infections, while younger FH individuals’ CVD risk is linked to thrombosis rather than elevated LDL-C.

## Data Availability

Not applicable.
